# Standing Breaks in Lectures Improve University Students’ Self-Perceived Physical, Mental, and Cognitive Condition

**DOI:** 10.3390/ijerph18084204

**Published:** 2021-04-15

**Authors:** Maike Paulus, Jule Kunkel, Steffen C. E. Schmidt, Philip Bachert, Hagen Wäsche, Rainer Neumann, Alexander Woll

**Affiliations:** 1 Institute of Sports and Sports Science, Karlsruhe Institute of Technology, 76131 Karlsruhe, Germany; maikepaulus@gmx.de (M.P.); steffen.schmidt@kit.edu (S.C.E.S.); philip.bachert@kit.edu (P.B.); hagen.waesche@kit.edu (H.W.); alexander.woll@kit.edu (A.W.); 2 Institute of Movement and Sport, University of Education Karlsruhe, 76133 Karlsruhe, Germany; rainer.neumann@ph-karlsruhe.de

**Keywords:** sedentary behavior, university students, standing break, health promotion

## Abstract

While adolescents and adults should limit high levels of sedentary behavior, university students spend large amounts of time on sedentary activities. The aim of this study was to investigate the effect of this prolonged sitting on students’ self-perceived physical, mental, and cognitive condition and to answer the question of whether simple standing breaks in lectures can help students improve these conditions and for example feel more concentrated, motivated, or less tense in class. A five-minute standing break was introduced using a designed presentation slide for one semester in five different 90-min lectures. In addition, an active break as well as an open break with no trigger were implemented in two further lectures to explicitly investigate the effects of a standing break. Before, during, and after the semester, the students were surveyed about their physical, mental, and cognitive condition (836 respondents at start, 634 during semester, and 528 at the end). To evaluate the practicality and acceptance of the standing break, lecturers were interviewed about their experience. At all survey time points, the standing break was highly accepted by the university students. About three quarters of the students felt a relaxation of the muscles in the neck and shoulder as well as in the back and the legs. More than three quarters perceived an increase in concentration, receptiveness and retentiveness, motivation, and well-being. Results of the statistical analysis indicate that a standing break as well as an active break are more effective than an open break to improve the self-perceived physical and psychological well-being of the university students. The increase in cognitive skills is reported by all groups, including the group who were offered open breaks. Hence, standing breaks in university lectures receive a high level of acceptance and practicability and have the potential to increase students’ physical, mental, and cognitive condition and contribute to students’ physical activity and health. While field research provides opportunities such as the testing of measures in the natural environment and producing real-life results relevant to the students and lecturers, it also imposes limitations as lecture settings differed, not all disturbances could be controlled, and the participation in the study might have led to social-desirability bias. For a sustainable development of a standing-friendly teaching and learning culture at universities, further interventions as well as the consideration of the topic in all processes and decisions within the universities are necessary. Since this study has taken place, student-life has changed drastically with COVID-19 measures. While this current paper is based on research conducted in 2019 and has only tested live lectures on campus, the tools tested could also be used for online lectures.

## 1. Introduction

Changes in people’s environment increasingly reduce the need for physical activity in everyday life. In conjunction with this development, people spend increasingly more time in sedentary activities [1]. According to the Sedentary Behaviour Research Network, sedentary behavior can be defined “as any waking behavior characterized by an energy expenditure of ≤1.5 metabolic equivalent (METs) while in a sitting or reclining posture” [2]. Despite the young research field, there is evidence that a sedentary lifestyle is associated with deleterious health effects, including an increased risk of cardiovascular disease, type 2 diabetes, metabolic syndrome, cancer and cardiovascular as well as all-cause mortality [3]. There are also indications that prolonged sitting can lead to muscular discomfort in the neck and shoulder area and the back [4]. In order to counteract the sedentary lifestyle, physical activity guidelines of many countries recommend limiting and interrupting prolonged sitting times as often as possible [5]. Thus, for the first time, the new 2020 WHO guidelines on physical activity also address the health impact of sedentary behavior and provide new recommendations on reducing sedentary behavior across all age groups [3,6]. The WHO strongly recommends that adults should limit the amount of sedentary time and ideally replace it with physical activity of any intensity for health benefits. In addition, adults should do more than the recommended levels of moderate- to vigorous-intensity physical activity to reduce the detrimental effects of high levels of sedentary behavior [3].

One of the first studies [7] in which breaks in sedentary time were objectively measured showed individuals with more interruptions in sedentary time had a significantly lower waist circumference and BMI and had significantly lower triglycerides and 2-h plasma glucose. In two other studies [8,9] with older overweight adults and young healthy adults, lower postprandial glucose and insulin concentrations were found when sitting was interrupted. In laboratory studies [10,11], frequent interruptions of prolonged sitting led to a higher energy level and reduction in musculoskeletal discomfort in the lower back. While a study [12] among ten healthy adults suggests that light-intensity activity breaks, but not standing, may enhance cardiometabolic health, it has been conducted with few people in a very controlled environment, where standing breaks were advised to be conducted as still as possible. Experts hence criticize that while evidence on sedentary behavior is often portrayed as nearly conclusive and guidelines have been prominently positioned, the sedentary behavior evidence base is still underdeveloped, incomplete, and inconsistent and cannot support quantitative guidance on sitting or the use of sitting breaks [5]. In a “narrative review of sedentary behaviour research paradigms and findings”, Stamatakis et al. [5] hint at the potential extra benefit of physical activity to break up sedentary time, as has been included in the 2020 WHO recommendations [3]. Ekelund et al. [13] have also concluded in a meta-analysis of data from more than one million adults that to eliminate the highest levels of sedentary behavior (which students would reach), one had to belong to the most physically active quartile, calling for more physical activity, advice that is also included in public health recommendations.

Effects of sedentary behavior on psychological outcomes, such as cognitive functions remain unclear. A systematic review of peer-reviewed literature [14] summarized that sedentary behavior is associated with lower cognitive performance, however only studies in the population of 40 years or older were considered. A further systematic review [15] related to work confirms the uncertainty and need for further retrospective, longitudinal, or epidemiologic studies. With the target group of middle-aged and older people, a cross-sectional study [16] of data of more than half a million adults investigated the connection between sedentary behavior and cognitive recline. While the authors do not describe their results for sedentary behavior per se, they point out the negative effects of certain behaviors, i.e., driving and television viewing. The latter example emphasizes the problem of sedentary behavior studies using other co-activities as indicators for sedentary time; in particular, as Stamatakis et al. [5] point out, the problem with confounding factors of TV “that are strong determinants of poor health outcomes but are not always accounted for”.

University students spend a lot of time in sedentary activities—more than their peers who are not students [17]. In addition, they are particularly prone to a sedentary lifestyle later in life [18], as sedentary time increases with higher academic degrees [19]. While studies using self-reports indicate that university students sit 7.29 h per day, objective studies are even higher (M = 9.28 h per day) [17], a relatively large margin of underestimation which is however not unusual [5]. In particular, university lectures lead to long sitting times among students. In a study conducted by Benzo et al. [20] with 993 students, 82.7% reported they would exclusively sit during the entire class time. Furthermore, almost all students (95%) preferred the possibility to stand during class and more than half felt that this could improve their health, attention, and feelings of restlessness. Some interventions have already been tested with the aim of reducing the time students spend sitting during university lectures. The introduction of sit–stand desks increased the standing time of students and led to improvements in engagement, participation and attention as well as declines in restlessness, fatigue, boredom, and cell phone use [21]. Another study [22] examined the effectiveness of active breaks which were instructed by trained students at the halfway point of lectures. Due to the active break, students felt a decrease in muscular tension and fatigue. Additionally, they reported an increase in concentration and well-being.

These findings support the need of further studies on interventions that interrupt students sitting time in lectures. Since active breaks require instructors or might not always be possible in lecture settings for other logistical or practical reasons, the aim of this study was to investigate the effect of more easily introduced standing breaks. As far as we know, there are no studies on the introduction of standing breaks in university lectures yet. This study of course also cannot fill the gap of an incomplete evidence base of sedentary behavior as an independent factor for chronic disease risk [5]. However, with the consideration of the critics’ remarks regarding the incomplete evidence base—and the extensive data required to fill this gap expected in the next four to five years [5]– this study provides another perspective on the problem and tackles the question of what effect sitting and sitting breaks have on the young people who are “stuck” in a sedentary setting. How does it make them feel and what can simple standing breaks deliver for the students as well as the lecturers?

## 2. Materials and Methods

### 2.1. Intervention and Participants

**Figure 1:**
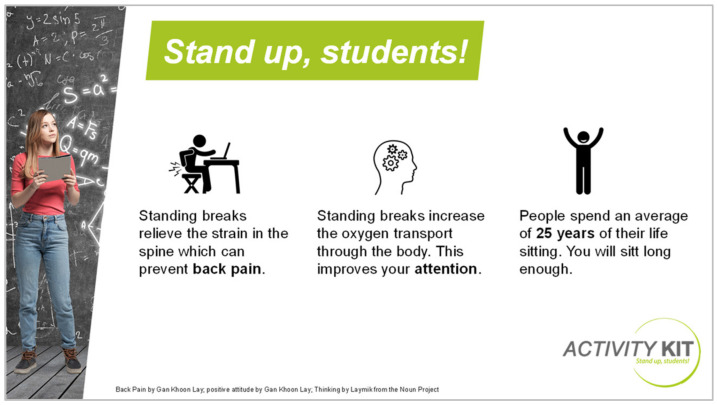
Presentation slide shown to the standing break intervention group as trigger to stand up.

The intervention took place in the summer semester of 2019 (from April to July) with the aim of implementing and evaluating standing breaks in university lectures. In order to find lecturers to participate in the study, e-mails were sent to various faculties at the university. As a result, the intervention was implemented in five lectures which took place once a week for one semester (14 weeks). Table 1 shows the participating lectures. The lectures differed in size while attendance also differed from one week to the next, as attendance was not mandatory and attendance lists were not recorded. The break was initiated each week by the lecturer at approximately half of the 90-min lecture by introducing a presentation slide about the advantages of standing into his or her presentation, which had been designed to encourage standing (see Figure 1). In order to explicitly investigate the effect of standing during the break, two further lectures were included in the study, both on the larger end of the scale. In one lecture for up to 303 students, five-minute active breaks with exercises for strengthening coordination, mobilization, and relaxation were introduced by a sports student while students in the other lecture for up to 760 students had an open break, with no trigger to stand or to be active. Five of the lectures took place in the largest lecture halls of the university, with up to 760 seats in fixed narrow rows. Two of the lectures were held in rooms for 20 and 90 students with moveable rows of chairs and tables. Table 2 provides an overview of the study groups.

### 2.2. Study Design and Data Collection

**Figure 2:**
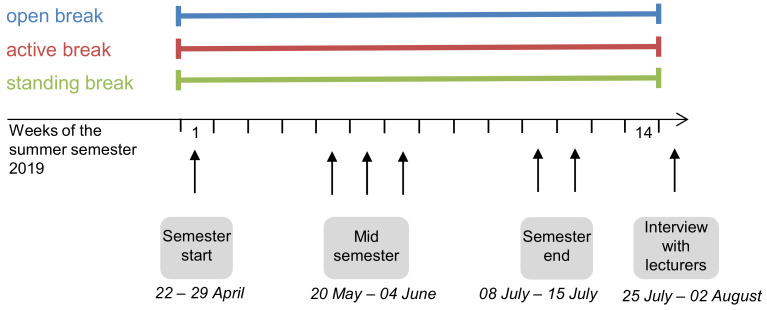
Study design with all study groups and the survey time points.

The efficiency of the intervention was examined with three online questionnaires for students of the lectures and took place at the beginning (week 1), during (weeks 5–7) and at the end of the semester (weeks 11–12). The online questionnaires could be accessed via a QR-Code or link and were completed during the five-minute break. In addition, lecturers of these lectures were interviewed on completion of the semester (week 15). Figure 2 shows an overview of the study design. Before the interviews were conducted, students and lecturers were informed about the purpose of the study and the anonymity of the data. Students and lecturers were not offered incentives to participate in the study.

### 2.3. Questionnaires

Questions at the start of the semester included sitting behavior during university courses and students’ physical, mental, and cognitive condition during long periods of sitting (≥60 min). The second and third survey focused on the introduction of the standing, active, or open break. Questions were asked about students’ behavior during the break and their physical, mental, and cognitive condition during the (standing, active, or open) break. The classification of the items to the categories physical, mental, and cognitive condition is shown in Tables 4 and 5. The questions about the physical, mental, and cognitive condition could be answered with a four-point Likert scale (certainly yes, rather yes, rather no, certainly no). The items were based on preliminary interviews with students and previous studies about the effects of long sitting periods as well as standing and active breaks [10,11,14,20,21,22].

### 2.4. Statistical Analysis

The programs SPSS (SPSS Statistics for Windows, Version 24.0 Armonk, NY, USA) and Excel (Microsoft Excel 2016 for Office 365) were used for data preparation and analysis. The collected data were analyzed on frequency distribution data. Answers to open questions were divided into categories. The second and third survey started with the question how often the person had attended the lecture and the intervention since the beginning of the semester. Students attending the lecture for the first time were excluded from the analysis, as were students who did not complete the questionnaire. To determine differences between the groups regarding the effect of the interventions, the Kruskal–Wallis test was used. Statistical significance was set at *p* ≤ 0.05. In the case of significant results, the Dunn–Bonferroni test was used as post hoc test to identify which groups differed significantly.

## 3. Results

### 3.1. Participant Characteristics

The sample consists of 836 university students at the start of the semester (S_start_), 634 university students at the second survey (S_mid_), and 528 at the third survey (S_end_). Table 3 shows the complete list of numbers of participating students in the different groups. Since there is almost no difference in the distribution of the survey sample regarding the desired degree, the number of semesters, and the students’ major between the survey time points, average values are given. The students were enrolled in the following degree programs: mechanical engineering (53.4%), mechatronics and information technology (9.7%), mathematics (7.4%), industrial engineering (7.0%), teaching mathematics (3.6%), and computer science (3.4%). Other degree programs (8.1%) were technical economics, business mathematics, educational engineering, teaching science and technology, information management, physics, and techno-mathematics. While 86.3% of the university students pursued a bachelor’s degree, 12.9% were enrolled in a master’s degree and 0.8% were studying abroad or for a doctoral or school study program.

### 3.2. Current Sedentary Situation during University Courses

In the following percentages, the options “certainly yes” and “rather yes” were grouped together. At the start of the semester, almost all students in the intervention groups (96.2%) indicated that they usually spend the entire lecture (90 min) in a sitting position. Only 2.1% of the respondents’ interrupted sitting with standing breaks. Stretching exercise (sitting or standing) was performed by 14.6% of the students and 4.1% indicated that they leave the lecture because of the long sitting time. Due to long periods of sitting (60 min or longer), more than half of the students reported feeling muscular tension in the shoulder and neck area (64.4%) as well as in the back (67.6%). More than three quarters of the respondents (78.8%) confirmed inadequate legroom while sitting. Knee pain is experienced by 22.9% and headaches by 23.1% of the students. Regarding cognitive abilities, more than three quarters of the students reported a decrease in concentration (87.9%), receptiveness (83.3%), and memory retention (76.4%). Furthermore, 84.5% felt a decrease in motivation, 86.7% an increase in fatigue and 59.0% an inner restlessness due to long periods of sitting.

### 3.3. Introducing a Standing, Active, or Open Break

For all three survey time points (S_start_, S_mid_, S_end_), the standing and active break as well as the open break were considered sensible by the majority of the students. The active break achieved a high approval with 95.4% at S_start_, 95.3% at S_mid_, and 93.1% at S_end_. The approval of the standing break increased after the start of the intervention, as it was considered sensible by 75.9% of students at S_start_, 87.0% at S_mid_, and 89.4% at S_end_. The open break reached the lowest rates of approval, although the rates were increasing, with 67.4% at S_start_, 71.4% at S_mid_, and 80.3% at S_end_. Both at mid-semester and at the end of the semester, more than 65% of all participating students report that they have attended every lecture, which indicates that the majority participated in the intervention several times. In lectures with the standing presentation slide almost all students used their breaks to stand up (93.1% at S_mid_; 94.4% at S_end_). In addition, almost a quarter of the students used the time to move around the lecture hall (16.4% at S_mid_; 24.5% at S_end_) or do stretching exercises (22.0% at S_mid_; 24.5% at S_end_). In the lecture with the open break and without a trigger to stand or to be active, about three quarters of the students indeed reported spending the break in a sitting position at both survey time points (81.7% at S_mid_; 74.6% at S_end_). However, 17.5% of the students who were “only offered an open break” reported using the break at S_mid_ to stand up which increased to 34.4% at S_end_.

Regarding the physical condition, about three quarters of the students taking a standing break felt a relaxation of the muscles at both S_mid_ and S_end_ (see Table 4 and Table 5). The relaxation of the muscles in the back was experienced most frequently with 83.3% at S_mid_ and 85.4% at S_end_. A similar result was found for the students of the active break intervention. The relaxation of the muscles in the neck and shoulder area was confirmed most frequently with 88.2% at S_mid_ and 90.2% at S_end_. In the control group, less than half of the students reported a relaxation of the muscles at S_mid_. At S_end_, this percentage increased to 54.1% for the neck and shoulder muscles, 56.6% for the back muscles, and 49.2% for the leg muscles. In terms of cognitive abilities, more than three quarters of the students taking a standing break experienced an increase in concentration, receptiveness, and retentiveness at both survey time points. The increase in the ability to concentrate was most frequently reported with 88.6% at S_mid_ and 91.1% at S_end_. Additionally, in the active break group as well as in the control group, more than three quarters of the participants experienced an increase in cognitive abilities. The increase in the ability to concentrate was also most frequently confirmed.

In the category of psychological well-being, more than three quarters of the participants in the standing break group felt more balanced, motivated, and awake at both survey time points. In addition, the standing break contributed to the well-being of more than 80% of the study participants. Among the study participants who took an active break, more than 80% stated that they are more balanced and motivated. More than 90% felt more awake and an increase in well-being. In the control group, less than 80% of the students felt more balanced, motivated, or awake at both survey time points.

More than half of the students who attended a lecture with the standing break (53.3%) reported that the intervention inspired them to interrupt sitting more frequently in other situations. Examples include when traveling to university by train as well as studying at their desk at home or the library. Of the students taking an active break, only 23.5% were inspired to interrupt sitting more frequently out of class. In all groups, more than 80% of the students felt that it was not noticeable that five minutes less were available for teaching. For the future, more than three quarters of the students in all groups would like to have regular standing, active, or open breaks in courses. The continuation of active breaks was the most frequently approved by 93.1% of respondents. In lectures that include a standing break, 87.4% would like to see the continuation. The continuation of open breaks was requested by 82.0% of the students in the control group.

### 3.4. Lecturers’ Opinion on the Standing Break

Five lecturers took part in an interview. Regarding the procedure and the length of the standing break, five minutes was sometimes perceived as too long. One potential solution mentioned by the lecturers is to shorten the standing break to two or three minutes or use it for discussions and questions. All lecturers found it difficult to start the standing break exactly at halftime of the lecture. For the future, they commented that the lecture presentation slides, and the content of the lectures should be better adapted to the standing break. Concerning the effects of the standing break, lecturers perceived the students as being calmer and more concentrated. Some lecturers found it very pleasant to have a break themselves between speaking, while others reported getting out of their flow. In some lectures, the five minutes needed to be compensated by teaching the content more quickly or by transferring it. However, in other lectures the standing break did not have any influence on the content. All lecturers stated that they will continue the standing break in future lectures. Generally, the lecturers considered it important to establish a standing-friendly environment in the university culture to implement standing breaks in all lectures.

### 3.5. Difference between the Standing, Active, and Open Break

In the category of physical condition, all items showed highly significant results at both survey time points (see Table 6). Regarding relaxation of the muscles in the neck and shoulder area, the Dunn–Bonferroni test indicated a significant difference between both the intervention groups (see Table 7). For the relaxation of the muscles in the back, results were highly significant for the difference between the intervention groups and the control group at both survey time points. Regarding the relaxation of the muscles in the legs, there was a significant difference at both survey time points between the intervention groups and the standing and control group. In the category cognitive abilities, at both survey time points significant results could only be found between the study groups for the impairment of the ability to concentrate and the impairment of. In the category mental well-being, the items balance, vigilance and increase in well-being presented significant results between study groups. For the item balance, the Dunn–Bonferroni test showed a significant difference between the standing break group and the control group at both survey time points. For the items vigilance and increase in well-being, significant group differences exist at both survey time points between the intervention groups and the control group (see Table 6 and Table 7).

## 4. Discussion

To our knowledge, this is the first study that examines the implementation of standing breaks in university lectures to break up students’ sitting time. The findings of the study support previous research reporting that students spend most of their lectures at university in a sitting position—which we expect to be even more nowadays with measures taken to control the COVID-19 pandemic. Due to long periods of uninterrupted sitting, more than half of the students felt muscular tension in the neck and shoulder area as well as in the back. Reasons for this may include the fact that more than three quarters of the students reported inadequate legroom while sitting, which can lead to an ergonomically harmful sitting posture. In addition, more than three quarters felt an impairment in their ability to concentrate, be receptive and retentive. Students also reported a decrease in motivation alongside becoming more tired. Similar results can be found in the study by Hosteng, Reichter, Simmering, and Carr [23], where classroom sitting time of college students was associated with a significant increase in discomfort and sleepiness.

We cannot deduce a causal relationship between prolonged periods of sitting and the impairment of cognitive abilities, an increase in fatigue, or decrease in motivation. However, it can be assumed that there is a multi-causal relationship, in which long and uninterrupted periods of sitting seem to be a factor.

As active breaks in lectures are not always feasible or desired by the lecturers and may impose a larger hurdle, the aim of this study was to investigate the effect of simple and easily implemented standing breaks on the students physical, mental, and cognitive conditions. The introduction of standing breaks after the first half of class time (approx. 45 min) led to many positive effects among the university students. These findings are consistent with a study by Jerome, Janz, Baquero, and Carr [21], where university students had access to sit–stand desks during class. The implementation of standing breaks led to an increase in attention and a decrease in restlessness for more than a half of the students. More than a third felt improvements in focus and engagement and a decline in fatigue and boredom.

The comparison of the standing break with an active and an open break provides evidence that taking a standing or active break during lecture is more effective than an open break in improving the physical condition and mental well-being of university students. Only a few studies compared the effect of uninterrupted sitting or standing breaks on changes in physical, mental, and cognitive conditions. The research group led by Bergouignan [10] found that test persons who regularly interrupt their sitting time experience less fatigue than test persons who sit continuously. In terms of cognitive abilities, no significant differences could be identified. The same result had been obtained in a study by Thorb, Kingwell, Owen, and Dunstan [11], which examined working at sit–stand desks. Test persons who interrupted their sitting by using a height-adjustable desk felt significantly more vigilant than test persons sitting at conventional desks. Regarding the implementation of a standing break and an active break, no differences could be found for the effects on students’ cognitive abilities and psychological well-being. There are significant differences between the intervention groups in the category physical condition, but neither the standing break nor the active break seemed more effective for relaxing all muscle groups. Although other studies [12] report positive objectively measured outcomes for bouts of physical activity and not for standing breaks, our study indicated that standing as well as active breaks could improve those conditions which are relevant to the students. In fact, regarding cognitive abilities, even open breaks can already help students feel more concentrated, motivated, etc. for the second half of the class.

Overall, the standing break was highly accepted by the university students. Likewise, many lecturers were open to the idea of standing breaks. More than half of the students participating in standing breaks reported that they also interrupt sitting more often during learning in the library, at home or in the office, as well as during train rides. However, only less than a quarter of the active break group do this, possibly as they may always associate a break from sitting with exercises rather than a simple standing break. This result highlights the potential that simple changes in conditions in university lectures can also influence students’ behavior off campus. Considering comparable positive results of active and standing breaks together with the easiness of the implementation of the standing break and the fact that students export it off campus supports the implementation of standing breaks in university lectures.

### Strengths, Limitations and Future Directions

Considering the simplicity of introducing standing breaks with one presentation slide, the importance to break up long sedentary times in university students, and its various positive effects which could be found, this study provides an easy solution for a substantial negative lifestyle factor of our time. One weakness of our study is the differences in setting between university lectures. These took place at different times and in different rooms, which can affect students’ perceptions. Furthermore, the active break had already been carried out and evaluated in previous semesters. Therefore, several students in the active break group were already familiar with the concept, which potentially led to contamination bias. In addition, the active break took place twice a week while the standing and open breaks were held only once a week. There is also evidence that the open break was not carried out strictly every week. These weaknesses are due to field research, where it is more difficult to control disturbance variables compared to laboratory research. However, the advantage of field research is to evaluate the interventions in students’ natural environment so that the results have a high external validity or relevance outside the present evaluation. The fact that students participated in the intervention and were thus aware of the focus of the study, might have led to social-desirability bias. Future studies should ensure that lectures take place at the same time and in the same rooms to improve comparability between the groups. In addition, the frequency of standing and active breaks as well as open breaks should not differ. If possible, standardized questionnaires should be used or the items about physical, mental, and cognitive condition should be operationalized. The inclusiveness of the different breaks for students with chronic conditions or disabilities was not a focus of this study. Consultations with the university’s representative for students with disabilities and chronic diseases support the integration of standing breaks as there would always be individuals who remain seated and attention would not be attracted by those who cannot participate in an active break (e.g., if one does not want to communicate a chronic disease). Taking a simultaneous break for the whole lecture would be preferred over standing individually during the lecture whenever students please to, as this commotion can pose challenges for students with hearing aids or on the spectrum of autism.

## 5. Conclusions

The present study indicates how the implementation of standing breaks in university lectures can improve students’ self-perceived physical, mental, and cognitive condition. Exercises for strength, coordination, mobilization, and relaxation can be performed during the standing break but this is not necessary to create the desired effect. When students spend the break in a sitting position, their self-perceived cognitive abilities improve, but less so their physical and mental well-being. This indicates that a break itself already improves cognitive conditions. The introduction of standing breaks in university lectures presents an easy and effective way of breaking up students’ sitting time which does not need instructors. Therefore, standing breaks can be implemented in all courses at the university. One advantage over active breaks is that there are always some who do not want to and do not have to stand; as such, students with disabilities or chronic illnesses who cannot stand and be active and might not want to explain themselves could remain seated without attracting unwanted attention or questions. As students also transferred standing breaks to their everyday life, there is potential for the intervention to contribute to the general promotion of students’ physical activity and health. Therefore, further interventions in different university settings are necessary to expand the potential. For this, it is important to break out of social norms and to integrate the concept into a university’s guiding principles and culture in order to achieve long-term changes. While this research was conducted on Higher Education students, it could be extrapolated to any other sedentary profession or adolescent students. The result that standing or active breaks help students or workers become more focused, motivated, and feel better at the same time could also encourage workplace interventions, while widely implemented interventions at universities have the potential to produce more aware leaders of the future.

## Figures and Tables

**Table 1:** Lectures of study.

Time	08:00–09:30	11:30–13:00	14:00–15:30
**Monday**		Automotive Engineering Lecture hall, 300 seats	Mechanical Design for Chemical Engineers Lecture hall, 760 seats
**Tuesday**		Interactive Systems Seminar room, 56 seats	
		Market Research Seminar room, 90 seats	
**Wednesday**	Mechanical Design for Mechanical Engineers Lecture hall, 760 seats		
	Linear Algebra Lecture hall, 303 seats		
**Thursday**		Fluid Mechanics Lecture hall, 734 seats	
**Friday**	Linear Algebra Lecture hall, 303 seats		

Standing break, active break, open break.

**Table 2:** Intervention and control groups.

Intervention Group Standing Break (5 University Lectures)	Intervention Group Active Break (1 University Lecture)	Control Group Open Break (1 University Lecture)
Standing break in one lecture a week Presentation slide with advantages of standing breaks	Active break in two lectures a week Exercises for strength, coordination, mobilization, and relaxation	Open break with no trigger to stand up or to be active in one lecture a week Presentation slide with advantages of breaks

**Table 3:** Sample sizes by study group.

Group	Semester Start (S_start_)	Midsemester (S_mid_)	Semester End (S_end_)
Complete sample	N_start total_ = 836	N_mid total_ = 634	N_end total_ = 528
Intervention group standing break	n_start standing break_ = 506	n_mid standing break_ = 380	n_end standing break_ = 304
Intervention group active break	n_start active break_ = 152	n_mid active break_ = 127	n_end active break_ = 102
Control group open break	n_start open break_ = 178	n_mid open break_ = 127	n_end open break_ = 122

**Table 4:** Students’ subjective perception during the standing break, active break, or open break during the lectures at midsemester in % (N = 631).

Category	Group	Standing Break				Active Break				Open Break			
	Item	cy	ry	rn	cn	cy	ry	rn	cn	cv	rv	cn	rn
Physical condition	Relaxation of the muscle in the neck and shoulder area	28.3	44.4	21.2	6.1	46.5	41.7	10.2	1.6	19.0	29.4	30.2	21.4
	Relaxation of the muscle in the back	40.7	42.6	11.6	5.0	29.1	44.1	23.6	3.1	19.0	29.4	27.8	23.8
	Relaxation of the muscle in the legs	49.5	28.6	15.9	6.1	23.6	37.0	30.7	8.7	14.3	19.8	36.5	29.4
Cognitive condition	Increase in the ability to concentrate	55.0	33.6	7.9	3.4	59.8	33.1	5.5	1.6	45.2	42.9	4.8	7.1
	Impairment of the ability to concentrate	8.2	10.6	24.3	56.9	1.6	10.2	18.9	69.3	7.1	7.9	30.2	54.8
	Increase in the receptiveness	43.1	43.9	9.3	3.7	44.9	45.7	7.9	1.6	37.3	49.2	7.1	6.3
	Impairment of the receptiveness	9.0	11.6	26.2	53.2	3.1	11.0	22.0	63.8	3.2	8.7	35.7	52.4
	Increase in the retentiveness	27.2	48.7	19.3	4.8	16.5	59.1	22.0	2.4	23.8	55.6	15.9	4.8
	Impairment of the retentiveness	6.1	13.8	34.1	46.0	0.0	7.9	33.9	53.8	4.0	11.1	36.5	48.4
Mental condition	Balance	38.9	46.8	9.5	4.8	34.6	53.5	10.2	1.6	26.2	50.8	17.5	5.6
	Increase in motivation	38.9	41.0	15.1	5.0	41.7	41.7	13.4	3.1	32.5	41.3	19.0	7.1
	Vigilance	57.1	34.1	5.3	3.4	64.6	29.9	3.1	2.4	30.2	42.1	23.0	4.8
	Increase in well-being	47.9	44.4	4.5	3.2	45.7	44.9	7.9	1.6	33.3	48.4	12.7	5.6

Cy = certainly yes, ry = rather yes, rn = rather no, cy = certainly no; students attending the lecture for the first time at S_mid_ and S_end_ were not included in the analysis.

**Table 5:** Students’ subjective perception during the standing break, active break, or open break during the lectures at semester end in % (N = 526).

	Group	Standing Break				Active Break				Open Break			
	Item	cy	ry	rn	cn	cy	ry	rn	cn	cv	rv	cn	rn
Physical condition	Relaxation of the muscle in the neck and shoulder area	35.8	40.4	16.9	7.0	48.0	42.2	6.9	2.9	21.3	32.8	28.7	17.2
	Relaxation of the muscle in the back	42.7	42.7	9.9	4.6	34.3	45.1	15.7	4.9	20.5	36.1	27.9	15.6
	Relaxation of the muscle in the legs	50.3	29.5	14.6	5.6	26.5	40.2	23.5	9.8	21.3	27.9	30.3	20.5
Cognitive condition	Increase in the ability to concentrate	53.0	38.1	5.0	4.0	55.9	40.2	2.9	1.0	46.7	45.1	4.9	3.3
	Impairment of the ability to concentrate	7.9	9.6	23.2	59.3	2.0	3.9	32.4	61,8	4.1	11.5	37.7	46.7
	Increase in the receptiveness	44.7	44.7	7.9	2.6	51.0	42.2	4.9	2.0	40.2	51.6	6.6	1.6
	Impairment of the receptiveness	6.6	8.9	28.1	56.3	2.0	5.9	34.3	57.8	3.3	14.8	36.1	45.9
	Increase in the retentiveness	27.2	52.3	15.9	4.6	28.4	51.0	15.7	4.9	22.1	65.6	9.0	3.3
	Impairment of the retentiveness	6.0	9.6	34.8	49.7	2.0	5.9	34.3	57.8	6.6	9.0	45.1	39.3
Mental condition	Balance	40.1	43.4	12.6	4.0	42.2	53.9	2.0	2.0	27.9	48.4	13.1	10.7
	Increase in motivation	39.7	41.1	15.2	4.0	50.0	38.2	8.8	2.9	32.0	42.6	13.1	12.3
	Vigilance	56.0	36.8	4.6	2.6	70.6	26.5	2.0	1.0	34.4	32.0	21.3	12.3
	Increase in well-being	46.4	42.1	7.0	4.6	53.9	38.2	5.9	2.0	30.3	50.0	10.7	9.0

Cy = certainly yes, ry = rather yes, rn = rather no, cy = certainly no; students attending the lecture for the first time at S_mid_ and S_end_ were not included in the analysis.

**Table 6:** Results of the Kruskal–Wallis test at S_mid_ (N = 631) and S_end_ (N = 526).

Items of Subjective Perception	S_mid_		S_end_	
	χ^2^	*p*	χ^2^	*p*
Relaxation of the muscles in the neck and shoulder area	52.4	<0.01	37.4	<0.01
Relaxation of the muscles in the back	54.1	<0.01	38.7	<0.01
Relaxation of the muscles in the legs	95.6	<0.01	51.9	<0.01
Increase in the ability to concentrate	6.0	0.05	2.3	0.31
Impairment of the ability to concentrate	7.6	0.02	6.0	0.05
Increase in the receptiveness	2.1	0.35	2.4	0.3
Impairment of the receptiveness	5.3	0.07	4.7	0.1
Increase in the retentiveness	2.2	0.33	0.1	0.94
Impairment of the retentiveness	9.6	<0.01	8.1	0.02
Balance	9.0	0.01	12.9	<0.01
Increase in motivation	4.3	0.12	10.9	<0.01
Vigilance	46.1	<0.01	48.3	<0.01
Increase in well-being	12.4	<0.01	16.8	<0.01

Students attending the lecture for the first time at S_mid_ and S_end_ were not included in the analysis; χ^2^ = Chi-Square; *p* ≤ 0.05 = significant.

**Table 7:** Results of the Dunn–Bonferroni test at S_mid_ (N = 631) and S_end_ (N = 526).

Items of Subjective Perception	Pairwise Comparison	S_mid_		S_end_	
		z	*p*	z	*p*
Relaxation of the muscles in the neck and shoulder area	standing break–active break	4.2	<0.01	2.9	0.01
	standing break–open break	4.6	<0.01	4.4	<0.01
	active break–open break	7.2	<0.01	6.0	<0.01
Relaxation of the muscles in the back	standing break–active break	−2.5	0.04	−1.6	0.32
	standing break–open break	7.3	<0.01	6.2	<0.01
	active break–open break	4.0	<0.01	3.6	<0.01
Relaxation of the muscles in the legs	standing break–active break	−4.8	<0.01	−3.9	<0.01
	standing break–open break	9.5	<0.01	6.9	<0.01
	active break–open break	3.9	<0.01	2.1	0.09
Impairment of the ability to concentrate	standing break–active break	−2.7	0.02	−1.3	0.63
	standing break–open break	−0.1	1.0	−1.7	0.27
	active break–open break	−2.2	0.08	−2.4	0.04
Impairment of the retentiveness	standing break–active break	−3.1	<0.01	−1.8	0.19
	standing break–open break	0.8	1.0	−1.6	0.34
	active break–open break	−1.8	0.19	−2.8	0.01
Balance	standing break–active break	0.3	1.0	1.5	0.38
	standing break–open break	3.0	<0.01	2.7	0.02
	active break–open break	2.2	0.09	3.5	<0.01
Increase in motivation	standing break–active break	-	-	2.0	0.13
	standing break–open break	-	-	2.0	0.15
	active break–open break	-	-	3.3	<0.01
Vigilance	standing break–active break	1.2	0.39	2.5	0.04
	standing break–open break	6.0	<0.01	5.6	<0.01
	active break–open break	6.2	<0.01	6.6	<0.01
Increase in well-being	standing break–active break	0.2	1.0	1.7	0.43
	standing break–open break	3.5	<0.01	3.3	<0.01
	active break–open break	2.5	0.04	3.9	<0.01

Students attending the lecture for the first time at S_mid_ and S_end_ were not included in the analysis; z = standardized test statistics; *p* ≤ 0.05 = significant.

## Data Availability

The dataset generated for this study is available on request to the corresponding authors.
